# The Role of the Songbird Trade as an Anthropogenic Vector in the Spread of Invasive Non-Native Mynas in Indonesia

**DOI:** 10.3390/life11080814

**Published:** 2021-08-11

**Authors:** Vincent Nijman, Marco Campera, Muhammad Ali Imron, Ahmad Ardiansyah, Abdullah Langgeng, Tungga Dewi, Katherine Hedger, Rifqi Hendrik, K. Anne-Isola Nekaris

**Affiliations:** 1 Oxford Wildlife Trade Research Group, School of Social Sciences, Oxford Brookes University, Oxford OX3 0BP, UK; mcampera@brookes.ac.uk (M.C.); ahmadardiansy@gmail.com (A.A.); 2 Little Fireface Project, Cipaganti 44163, Indonesia; abdullahlanggeng@gmail.com (A.L.); tdhp25@gmail.com (T.D.); katey.hedger@gmail.com (K.H.); rifqihendrik@gmail.com (R.H.); 3 Faculty of Forestry, Universitas Gajah Madah, Yogyakarta 55281, Indonesia; maimron@ugm.ac.id; 4 Forest and Nature Conservation Policy Group, Wageningen University, 6708 PD Wageningen, The Netherlands; 5 Primate Research Institute, Kyoto University, Kyoto 484-8506, Japan

**Keywords:** Asian songbird crisis, alien invasive species, CITES, conservation, convention on biodiversity, Indonesia, wildlife trade

## Abstract

The wildlife trade has facilitated the introduction of invasive non-native species, which may compete with native species for resources and alter ecosystems. Some of these species have great potential to become invasive if released or escaped from captivity. Here we studied the pet trade in a group of open countryside birds, the mynas (*Acridotheres* spp.) in Indonesia, and identified the areas that are at high risk of facing the establishment of these species. Mynas are among the most invasive birds in Southeast Asia. Once established in a new area, they are almost impossible to eradicate and can have strong negative impacts on the ecosystem. Preventing their introduction is therefore essential. Yet, invasive non-native mynas continue to be traded openly. We present data on the trade in seven species of mynas on Java, Bali and Lombok, with three species being native to parts of one or two of these islands, but not to the remainder, and four that are non-native to the region. From 2016 to 2021 we conducted 255 surveys of 30 animal markets. We recorded over 6000 mynas that were offered for sale outside their native range. Areas most at risk because of their high prevalence in specific animal markets, are Greater Jakarta, eastern Java, Bali and Lombok. The number of invasive non-native mynas recorded was positively related to the size of the animal market. Indonesia is signatory to several international agreements (CBD, ASEAN) that have policies and guidelines to prevent the introduction of invasive non-native species, but compliancy is weak. Annually hundreds and possibly thousands of invasive non-native mynas are released by Indonesian conservation authorities in regions that are outside their native range. Effective management of, and regulation of trade in, potential invasive non-native birds in Indonesia falls short and inadvertently greatly aids both their introduction and establishment.

## 1. Introduction

The global trade in wildlife has facilitated the introduction of species to new regions, where they can have negative effects on ecosystems and human societies. The international trade in wildlife has intensified in recent decades, both in number of species that are traded, the number of individuals that are traded and the number of countries that are involved [1]. This is especially true for the trade in ornamental birds in Southeast Asia [2,3,4,5]. Several studies explicitly address how this trade facilitates the introduction of invasive non-native species [6,7,8,9,10,11]. We here use the term ‘invasive non-native species’ (also referred to as ‘invasive alien species’ or just ‘invasive species’) to refer to species that have been introduced, accidentally or intentionally, outside of their natural geographic range and that have interfered with the native resident species [12,13,14]. These species are often introduced as a result of the globalisation of economies through the movement of people and goods [14]. We use the word ‘native’ in a narrow sense, referring specifically to a geographic region. This may or may not coincide with political borders. Thus, a species can be native in the westernmost part of a country, but when it is introduced to the easternmost part of that country it can be considered an ‘invasive non-native species’, even though it has not crossed any international borders.

When considering the potential for successful non-native species invasion, i.e., long-term establishment, it is important to take into account its biological characteristics as well as the environmental conditions of the area where it may become established. Ewel [15] noted that “species invasions often reflect the conditions of the community being invaded rather than the uniquely aggressive traits of the invader”.

For birds, it has been argued and demonstrated empirically, that in terms of effective management, any proposal to ban the trade in wild-caught birds could reduce the spread of invasions already underway and to prevent future ones [16,17]. The common myna *Acridotheres tristis* is a species that ranges in Asia and has been introduced in many parts of the world. It is one of only three bird species included on the list of ‘100 most invasive species’ [18,19,20]. Yap and Sodhi [21] recognised two additional species of myna as among the 16 most invasive bird species in South-East Asia. These are the Javan myna *A. javanicus*, from Java and Bali that had been introduced via the cage bird trade into Singapore and from there expanded into West Malaysia, and the crested myna *A. cristatellus* from China and Indochina that had been introduced into West Malaysia and the northern Philippines, partially as a biological pest control and through the cage bird trade. 

Building on this, we argue that those species that are traded in larger numbers outside their native range will have the potential to result in a larger introduction effort, either through deliberate releases or through accidental escapes. Using data on the trade in seven species of mynas on the Indonesian islands of Java, Bali and Lombok, we gain novel insights in the potential risks that the trade in these species poses for the introduction of invasive non-native species. In Indonesia, keeping wild-caught songbirds in cages, to admire their plumage and/or their song is very common [22,23,24]. Among the people of Java (including the Betawi, Sundanese and Javanese, both living on Java and other islands) the keeping of a bird in a cage is part of a balanced life as it represents time devoted to a hobby [25]. What bird species someone keeps reflects, in part, their social-economic status [26], with less common (including non-native) birds demanding a premium (i.e., being more expensive). 

The three study islands are anthropogenically disturbed (more than 90% of its original habitat has been replaced by human-modified landscapes: [25]) and are home to some 140 million people. Areas that were previously covered in closed canopy forest are replaced by more open forest types or even open savannah-type vegetation. This may result in areas that previously were unsuitable for specific invasive species to become fitting, but it also can reduce the impact of already present invasive species to the extent that they may no longer be invasive [27]. Especially the lowlands of Java and to a lesser extend Bali and Lombok have a lowered diversity of native species, either because of extirpation or extinction [28] or because of excessive trapping [29], thus, inadvertently creating vacancies for invasive non-native species (see also [30]). As such, large parts of Java, Bali and Lombok offer favourable conditions for invasive non-native bird species to establish. 

Using novel data from a large-scale animal market survey we created four specific predictions, namely:

1. Animal markets are found all over the three islands and therefore all geographic regions are equally likely at risk from invasive non-native mynas. As a null hypothesis we expect the numbers of invasive non-native mynas in trade to be similar in all regions. If this turns out not to be the case, from a management perspective this allows the authorities to focus their efforts more to those areas that are most at risk.

2. While we do not expect all invasive non-native mynas to be traded in equal numbers, we do not expect any particular species combinations to be traded alongside each other.

3. We included a wide range of animal markets in our surveys, from small to very large, that are situated in cities differing in size. We do not expect any clustering of animal markets to occur with regards to the number of non-native mynas or with regards to specific species compositions.

4. Mynas are among the most commonly traded songbirds in cities with different levels of affluence. We do not expect any relationship in the number of invasive non-native mynas offered for sale and affluence, measured as the government recommended minimum monthly wage for the year 2020.

Based on our findings, we discuss the policies that Indonesia has in place for preventing or minimising the risk of the establishment of invasive non-native species. 

## 2. Materials and Methods

### 2.1. Selection of Potentially Invasive Mynas on Java, Bali and Lombok

Mynas are generalist species with high reproductive rates and relative short generation times, they are long-lived and have high dispersal rates; within their native range all species we consider occupy a broad niche (habitat, diet, nest site selection) [31]. There are a number of traits that have made mynas successful invaders, including, like all songbirds, relatively large brain sizes (thus increasing survival in novel environments), moderate levels of aggression, broad diets, and good dispersal abilities [32,33]. When established they compete for nest cavities and because of their high levels of aggressive behaviour displace other species. Mynas can also be considerable agricultural pests, especially causing damage to ripening fruits. Hybridisation is a problem for at least black-winded, grey-backed and grey rumped mynas [34].

We consulted the Global Register of Introduced and Invasive Species (www.griis.org, accessed on 11 August 2021) and selected ten candidate species of mynas as part of our survey. They included seven species that range outside the study area but that either have been observed in trade in the study region (e.g., [24,35,36,37]) or that have established themselves as invasive non-native species in insular Southeast Asia (e.g., [20,38,39]). The other three species occur naturally in parts (but not all) of our study area of Java, Bali and Lombok. Thus, the crested myna is considered an invasive species on all three islands and the black-winged myna in the easternmost part of Java, Bali and Lombok, but not on most of Java. The Java myna is not considered invasive on Java or Bali, but it can be on Lombok. Seven of the ten candidate species recorded by us in the animal markets and the focus here is on these species (Table 1).

### 2.2. Data Acquisition—Animal Market Surveys

**Figure 1:**
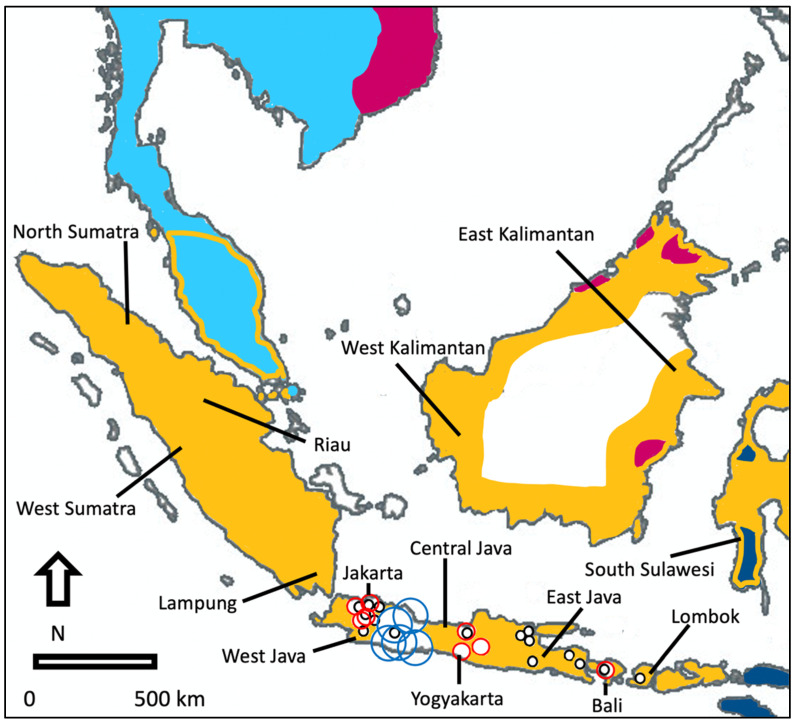
Distribution of myna species in parts of Southeast Asia (including areas where they are newly established) and animal market survey effort plus the locations of all provinces mentioned in the text. Dark blue: pale-bellied myna; light blue: common myna; purple: crested myna; yellow: Javan myna. Small black circle: animal market that was surveyed between three and five times, intermediate red circle: animal markets that were surveyed between six and fifteen times; large blue circle: animal markets that were surveyed sixteen times or more.

Between August 2016 and February 2021, we surveyed animal markets on Java, Bali and Lombok, Indonesia. Locally these are referred to as *pasar hewan* (a market where mainly domestic animals are traded, alongside wild-caught ones), *pasar satwa* (a market where mainly wild animals are traded, alongside domestic ones) or *pasar burung* (a market where domestic and wild-caught birds are traded, alongside a smaller number of animals that are not birds). We here focus on 30 animal markets that were each surveyed at least three times or more, with at least one month between surveys; we refer to these as ‘monthly surveys’ as they are at least a month apart, but we acknowledge that for most market surveys the interval is longer (Figure 1). The total number of monthly surveys in these markets was 255. The animal markets are open to the public and all apart one (Pon, Semarang) are open all days of the week from early morning to early evening. Animal markets range from the three-story Pramuka market in Jakarta, with over 200 shops that require most part of a day to survey, to much smaller markets with around ten shops that can be surveyed in less than an hour. 

Traders openly sell all types of birds in the animal markets, including protected species that should not be traded, regulated species for which only a limited number of individuals can be traded, and ones that are not subject to any regulations (e.g., captive-bred non-native species). Typically, one or occasionally two surveyors walked through markets slowly, recording numbers of individual birds in each animal market and species in mobile phones or by memorizing numbers and recording the data in a notebook directly after having left the market. We did not survey back alleys. We did not purchase any birds or other wildlife.

### 2.3. Analysis

We only included surveys that were at least a month apart in the analysis; the mean survey interval for individual markets was three months and six days. We assumed that mynas observed in the various animal markets are different individuals, i.e., we assume that birds do not move between shops, between markets or between cities. We expect that all mynas that were observed in subsequent surveys were different birds. Nijman et al. [13] found a high turnover of at least 70% after two weeks for black-winged mynas in seven of these animal markets and it is therefore reasonable to assume that indeed after more than three months the mynas have been sold. 

For each city we calculated the mean number of invasive non-native mynas for each survey. Comparing this to all mynas we observed in trade, including native ones, we calculated for each city the proportion of invasive non-native mynas for sale. We then pooled this by province (i.e., Jakarta, West Java, Central Java, Yogyakarta, East Java, Bali and Lombok) and used Chi-square tests to test whether or not there are geographic differences in the numbers for sale. 

The test whether or not specific species combinations are traded alongside each other, we created a presence-absence matrix for the seven species and 29 wildlife markets with at least one detection and created a heatmap using ClustVis [40]. ClustVis uses several R packages internally, including ggplot2 for the principal component analysis (PCA) plot, pheatmap (R package version 0.7.7; Free Software Foundation, Boston, MA, USA) for plotting heatmap and PCA. We used the default setting for the PCA analysis, i.e., singular value decomposition with imputation. For data pre-processing the unit variance scaling method divides the values by standard deviation so that each row has variance equal to one [40]. 

Using the same presence-absence matrix and using ClustVis we tested if animal markets clustered by province or by size, i.e., animal markets in e.g., Jakarta are grouped together as are the ones in East Java or very large animal markets cluster with other very large animal markets. Animal markets may change in size between weekdays and weekend, with more shops open during the weekends, and over the course of the 4.5 years study shops may close whereas others open. As such, we considered the size of the market as an ordinal variable from one (small) to four (very large) as in this way size classes were more robust.

We ran a generalised linear mixed model to test whether the number of invasive non-native mynas (mean number per market, for all non-native species combined) offered for sale in the 30 markets were dependent on the size of the market, the Indonesian government recommended monthly minimum wage (*upah minimum 2020*, converted to US dollars) and the human population in the regency or city (in case of large cities). We divided the data by year and market and considered the mean number of mynas detected in each market each year (rounded at the closest integer) as statistical unit. We used the market and year as random effects and the log10 of the number of market surveys in the market in a certain year as weight in the analysis. We used the package “glmmTMB” for the analysis and considered different fit families for count data (nbinom1, nbinom2, poisson, genpois, compois) available in the package [41]. We also included or excluded a zero-inflated factor in the model and tested the model diagnostics for model selection via the package “DHARMa” [42]. We selected the model based on QQ plot residuals and residual vs predicted plot, considering the only model with no significant problems (i.e., Poisson fit with no zero-inflation). We ran the analysis in R (Version 4.0.4; Free Software Foundation, 51 Franklin Street, Fifth Floor, Boston, MA 02110, USA), and we accept significance when *p* < 0.05. 

## 3. Results

### 3.1. Invasive Non-Native Mynas in Trade

Focusing only on animal markets where specific myna species can be considered invasive, we recorded over 6000 mynas of seven species, i.e., 5569 common mynas, 301 crested mynas, 402 Javan mynas, 95 black-winged mynas, 74 grey-rumped mynas, 12 bank mynas, and 3 pale-bellied mynas (Table 2). The highest mean numbers recorded per survey were ~140 to 175 for common myna, ~100 for Javan mynas, but below 20 for crested and black-winged mynas. We did not record any grey-backed mynas *A. tertius*, endemic to Bali and Lombok, although the latter is difficult to distinguish from black-winged and grey rumped myna when young and many younger individuals of the former were observed in trade. 

**Figure 2:**
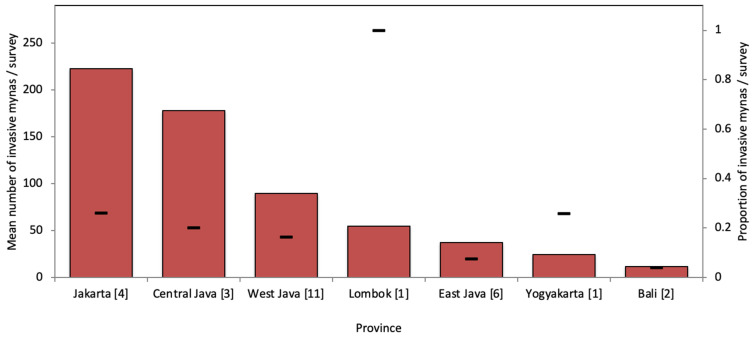
Invasive non-native mynas offered for sale in seven provinces in Indonesia (with the number of markets included in each indicated between brackets), showing the mean number of invasive non-native mynas per survey in each province (bars) and the proportion of invasive non-native mynas of the total number of mynas for sale (dash).

The number of invasive non-native mynas that are offered for sale on any given day range from ~200 in Jakarta and Central Java, to ~100 in West Java and ~50 in East Java and Lombok (Figure 2). Even when corrected for the number of animal markets that were surveyed in each of these provinces, the number of invasive non-native mynas for sale differs geographically (χ^2^ = 123.56, df = 6, *p* < 0.0001). Cities that stand out because of their high number of invasive non-native mynas offered for sale are Jakarta and Surakarta on Java and Mataram on Lombok (Table 2). The proportion of invasive non-native mynas for sale is particularly high (>25%) in Jakarta, Yogyakarta, and especially Mataram on Lombok (Figure 2).

**Figure 3:**
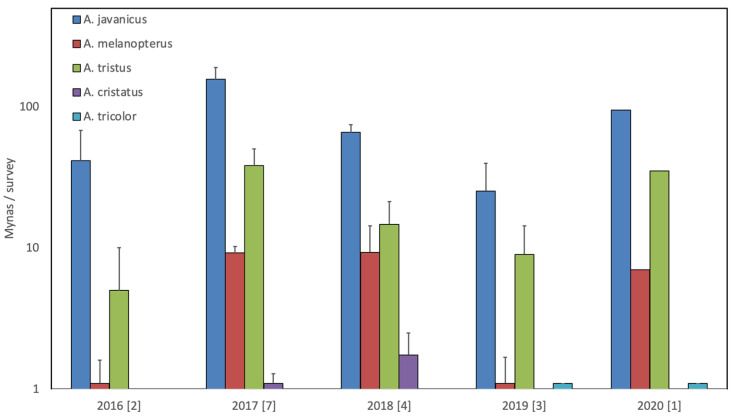
Number of mynas (mean + one standard error of the mean) recorded during surveys of Plered bird market in Cirebon, West Java during 17 surveys over five years (number of surveys per year is given between brackets). Two non-invasive species, i.e., *A. javanicus* (blue) and *A. melanopterus* (red), and three invasive species, i.e., *A. tristus* (green), *A. cristatus* (purple) and *A. tricolor* (pale blue), were recorded. Note the logarithmic scale on the left.

Focussing on individual animal markets, e.g., Plered in Cirebon in West Java (Figure 3), show that the numbers that are offered for sale differ markedly for the various species, but various species are consistently available at the same time, and in all years three to four species, including two invasive species, are present. 

**Figure 4:**
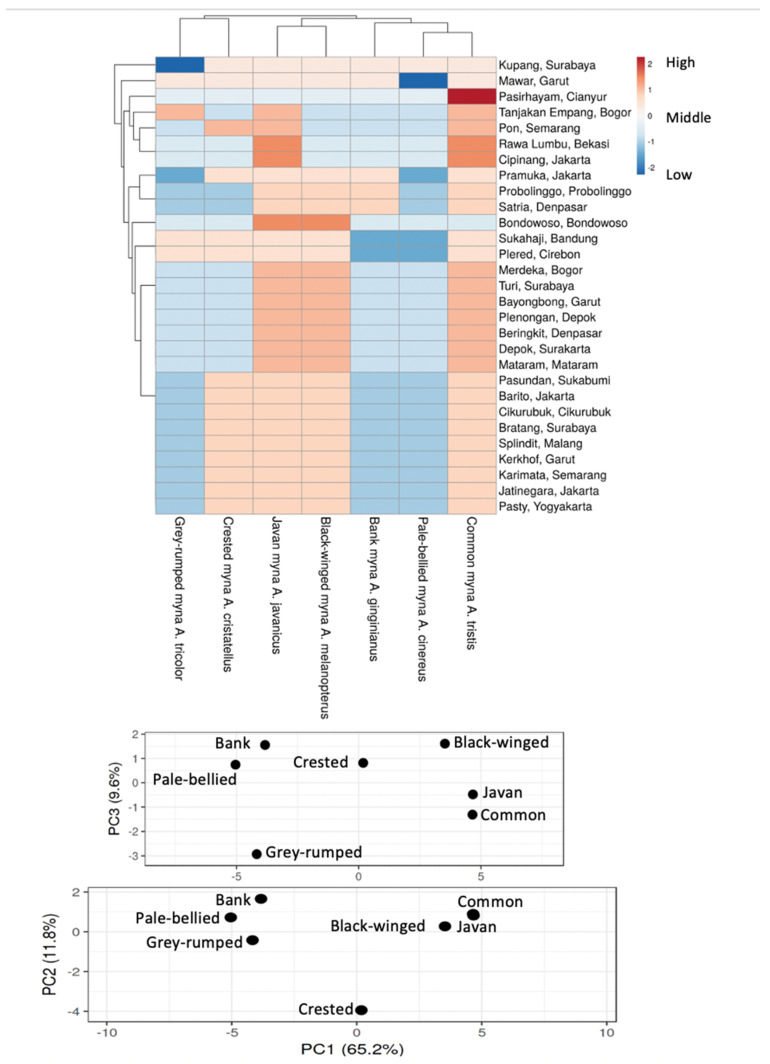
**Top**: Hotspot analysis for invasive non-native mynas in trade in Java, Bali and Lombok, Indonesia; the values are relative with red indicating the highest values and pale blue the lowest. The cladograms at the top represent the clustering of species whereas the cladogram on the left indicates the clustering of animal markets. **Below**: Results of the principal components analysis.

In the ClustVis heatmap analysis the first PCA axis explained 65.2% of the variation, the second explained 11.8% and the third 9.6%. Regressing PCA1 against PCA2 allowed for clear identification of most species, albeit that common and Javan mynas showed considerable overlap (Figure 4); this was resolved when regressing PCA1 against PCA3. 

When considering species, as illustrated by the dendrogram at the top of Figure 4, cluster analysis shows two clusters, one grouping grey-rumped with crested myna (invasive non-native species in most of the study region), and the second with the other five species. This second cluster then comprised of two branches, one grouping Javan with black-winged myna (non-invasive) and the other grouping bank, pale-bellied and common mynas (all invasive non-native species). This suggests that while two of the three non-invasive species are traded alongside each other, more so than the other species, there is no consistent pattern by which the invasive non-native species are traded. 

The clustering of animal markets (dendrogram on the left-hand side of Figure 4) was less easy to interpret with neither the geographic regions or market characteristics explaining the observed pattern. Animal markets did not group into provinces, nor did markets in the west group with other markets in the west and ones in the east with others in the east (for locations, see Table 1). Finally, large markets did not group with other large markets or small markets with other small ones. 

### 3.2. Determinants of Invasive Non-Native Mynas in Trade

**Figure 5:**
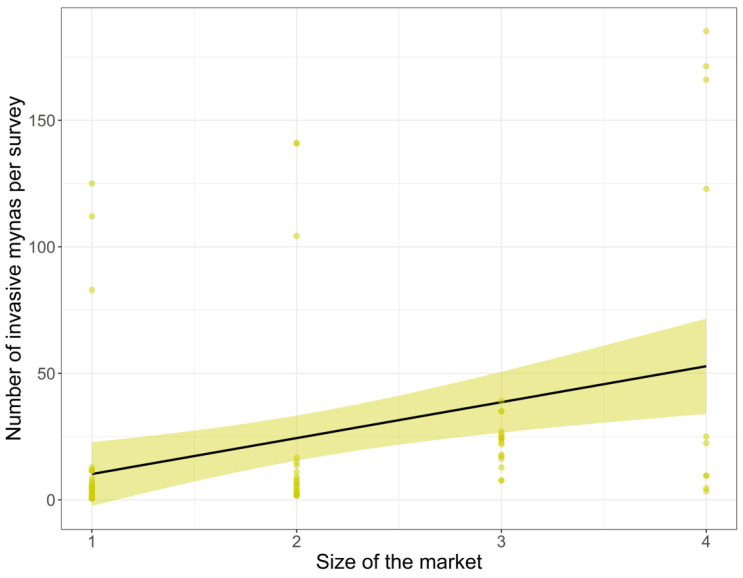
Significant results of the generalised linear model with the mean number of invasive non-native mynas offered for sale in 30 animal markets in Indonesia as response variable. Values are model predicted values, lines are model fit lines, shaded areas are 95% CI. Surveys were done between August 2016 and February 2021 (with surveys at least a month apart).

The number of invasive non-native mynas offered for sale in markets was positively related to the size of the market (estimate = 0.710 ± SE 0.267, z = 2.656, *p* = 0.008; Figure 5), and the variation was similar between years (random effect variance: 0.030). 

The minimum wage (estimate = 0.910 ± SE 1.127, z = −0.807, *p* = 0.420) and the human population size of the city or regency where the animal market was based (estimate = 0.049 ± SE 0.099, z = 0.368, *p* = 0.133) did not have a significant effect on the number of invasive non-native mynas species offered for sale (Figure 5).

## 4. Discussion

We tested four predictions to gain insight in the trade of invasive non-native mynas as to inform us about the potential threat they pose for establishing themselves on the islands of Java, Bali and Lombok. While we expected them to be traded equally in the various regions, we found the highest numbers in Jakarta and Central Javan and relatively low numbers in East Java and Bali. The proportion of non-native mynas was highest in Lombok, as there the one native species of myna was not recorded in trade but three non-native species were. As predicted, we did not find any clear co-occurences of myna species in trade. Species that are found in Indonesia were traded alongside ones from other parts of Asia.

With regards to the trade in invasive non-native mynas there are no apparent characteristics of animal markets that inform us about what species are traded where and in what quantities. Large animal markets have, on average, more individuals on offer, but when combining species and abundances, large animal markets are not more similar to each other than they are to smaller ones. 

The number of invasive non-native mynas for sale in a city is not correlated with the number of people that live in that city—more potential buyers does not affect their availability—nor it is related to its spending power. It is unlikely that buyers of invasive non-native mynas are a random subset of people living in a city. Rather they represent one or more specific socio-economic groups as indeed seen with other bird species in trade [22,26], and that this differs between cities. 

### 4.1. Invasive Non-Native Birds in Java, Bali and Lombok

Prime locations where invasive non-native birds manage to establish stronghold often include villages and cities, disturbed habitats and agricultural land [7,10,20,33,43,44]. Java, Bali and Lombok have little natural habitat (forests, woodlands, mangroves, etc.) remaining [25], and possess the environmental conditions for mynas to establish themselves as invasive non-native species throughout the region. With limited focussed research it is clear that while several species of myna have been recorded as invasive non-native species on Java, Bali and Lombok, details on numbers and their actual spread is lacking. This is an area that requires urgent attention. 

Several researchers have noted that most long-distance introductions of non-native species to new areas are the direct or indirect results of human activities, and social and economic factors are often as critical as biological factors in the introduction, and establishment of invasive non-native species [21,30,33,38]. The minimum straight-line distances between areas where we have observed specific invasive non-native mynas to their native habitats range from 30 km for the Javan myna, to 100 to 350 km for black-winged myna and 400 to 500 km for grey-rumped myna. For the species that are not native to our study region, these distances are larger, i.e., 600 km for pale-bellied myna, 1000 km for common myna, 3000 km for crested myna and 3700 km for bank myna. These distances clearly illustrate the efforts traders have gone to in order to obtain birds and equally points at the globalisation of the songbird trade.

The large numbers that we recorded in trade indicate that (a) mynas, both species that are native to Java, Bali and Lombok and ones that are not, are consistently present and (b) the volume of trade allows for repeat (accidental) introduction throughout the region. Wealth, amongst other factors, has been postulated as an important factor in explaining variation in where wildlife trade takes place and who participates. In this study we did not find relationship between invasive non-native mynas for sale in regions and government recommended minimum wages. The sales prices for some of these mynas are very low and even the more expensive species are still affordable for a large part of Indonesian society. The trade has a long history in various parts of Java. In the 1980s, Basuni and Setiyani [35] reported that Javan myna was the most commonly traded bird in Jakarta’s Pramuka bird market, with hundreds of them sold every month, and Djuwantoko [45] reported on the presence of hundreds of common myna and Javan myna in Yogyakarta’s Ngawi bird market and Semarang’s Karimata bird market.

The trade in birds in Indonesia is heavily regulated, at least on paper. Over a 100 native species are listed as protected and wild-caught individuals cannot be legally traded [46]; these include black-winged, grey-backed and grey-rumped myna. Second generation captive-bred offspring of these protected species can be traded but only when accompanied by appropriate paperwork. Wild-caught individuals of other native species can only be traded in limited numbers from specific regions, which are determined annually at a meeting organised by the Ministry of Forestry. In 2019, for example, midway during our study, the quotas were 4500 Javan myna from Sumatra and 100 Javan myna from Kalimantan (Indonesian Borneo) and 3900 common myna from Sumatra. While there are no restrictions on where these species can be traded, any movement between provinces within Indonesia need to be covered by appropriate permits. As such, these two species of myna can be traded in parts of Indonesia where they are not native, including islands in eastern Indonesia. There are no capture quotas for any other mynas within Indonesia, nor are there any provisions in the law to prevent of restrict the import and sale of non-native mynas.

### 4.2. National Policies and Strategies Concerning Invasive Non-Native Birds

Within Indonesia there is a lack of oversight for both the trade in invasive non-native birds such as mynas and their monitoring and ultimately their removal. Multiple governmental departments at different administrative levels are involved, with the local natural resource management agencies and the Ministry of Environment and Forestry assumed to have primary responsibility. The Ministry of Agriculture and the Ministry of Trade also bear some responsibility. Since 2000 centralized government and development planning has been replaced with a series of decentralization programmes. This has allowed for greater authority, political power, and financial resources to be handed over directly to regencies and municipalities, bypassing the provinces. As such, regents and mayors share the responsibilities with central government agencies. These lower-level responsibilities included trade, agriculture and the environment, areas of direct relevance for the management of invasive non-native species. However, the way in which these management responsibilities are delegated between the various (local and national) government departments is unclear. Furthermore, any required actions have to comply with multiple departmental regulations, some of which may be conflicting. 

The import of birds into Indonesia, and restrictions on this import, are mostly discussed in the context of the Convention on International Trade in Endangered Species of Wild Fauna and Flora (CITES), to which Indonesia acceded in 1978 [47], or, alternatively, in the context of human health and the risk of spreading Avian Influenza [48]. Invasive non-native species are relevant to CITES and are addressed specifically in its Regulation 13.10. CITES urges strong cooperation between CITES and the Convention of Biological Diversity (CBD). CITES recommends Parties to consult with appropriate bodies when considering the import of potentially invasive species. However, by its very nature, CITES almost exclusively deals with international trade in species that are listed on one of its three appendices [49]. Domestic trade and trade in non-listed species fall outside CITES’s remit. 

Indonesia has a harvest quota from specific provinces for a limited number of bird species [47]. Species that have not been allocated a quota cannot be traded commercially. In recent years, the Javan myna has only been allocated quotas in four provinces in Sumatra and one in Kalimantan, while the common myna has been allocated quotas for three provinces in Sumatra. This should preclude any harvest of mynas from Java, Bali or Lombok; we found many mynas in the animal markets and traders acknowledged that at least a proportion of them were locally sourced. 

The Indonesian authorities occasionally confiscate legally protected mynas in trade and as a rule these birds are released nearest to the area where they were confiscated or nearest to the offices of the arresting authorities. In 2018, following the seizure of 14 black-winged mynas, one bird was released in the easternmost part of Java and 13 were released on Bali; both these areas are outside the natural distribution range of black-winged mynas [34]. In 2020, after 1420 Javan mynas were confiscated in North Sumatra, the authorities released 1358 of them in the local 61-hectare small Dolok Tinggo Raja recreation forest [50]. In a separate event, 80 Javan mynas were released in Lampung [51]. That same year, authorities in East Kalimantan confiscated 282 Javan mynas, brought them over to South Sulawesi, where they were released in the Bacukiki protected forest [52]. In 2021, 32 Javan mynas that had been confiscated from two bird traders were released in West Sumatra, and another ten, confiscated from a third trader, were released in Riau [53,54]. In addition, in 2021, 174 Javan mynas were released in West Kalimantan [55]. These releases were conducted by the Indonesian Natural Resource Conservation Agency (BKSDA); North Sumatra, Lampung, East Kalimantan, South Sulawesi, West Sumatra, Riau and West Kalimantan are all provinces outside the natural range of the Javan myna. While this listing is far from exhaustive, combined these data suggests that, certainly in recent years, annually hundreds, or more likely, thousands of invasive non-native mynas are released deliberately. 

Indonesia is signatory to a number of agreements, that have articles, and for which clear statements have been made, relevant to invasive non-native species. These could aid in the prevention of the spread of mynas outside their native ranges in Indonesia. Indonesia is a Party to the CBD. Article 8(h) of the CBD states that “Each contracting Party shall, as far as possible and as appropriate, prevent the introduction of, control or eradicate those alien species which threaten ecosystems, habitats or species”. Directly linked to the CBD are the Aichi Biodiversity Targets, including Target 9: “By 2020, invasive alien species and pathways are identified and prioritized, priority species are controlled or eradicated, and measures are in place to manage pathways to prevent their introduction and establishment.” Indonesia is also a member of the Association of Southeast Asian Nations (ASEAN) and in 1985, this organisation adopted the ASEAN Agreement on the Conservation of Nature and Natural Resources. Following Article 3(3) this agreement requires that Parties endeavour to regulate and, as appropriate, prohibit alien species introductions. The international, as well as domestic, pet trade is likely responsible for a substantial part of the spread of invasive species; the large number of non-native invasive species that are purchased either escape or are released into the wild by the people that have bought them [6], or, in Indonesia, the authorities that have confiscated them.

In order to comply with these agreements in 2015 Indonesia published its National Strategy and Management Action Plan for Invasive Alien Species [56]. While sections of this strategy are generic and relevant for mynas as invasive non-native species, the majority of specifics presented in the strategy refer to terrestrial plants or aquatic animals. In fact, despite the massive and persistent songbird trade in Indonesia, birds are only mentioned twice, first referring to the country’s rich birdlife and second, in that birds can be a vector for invasive non-native species that affect human health. In terms of research, one of the few studies on invasive non-native birds that has been published from Indonesia, deal with grosbeak starlings *Scissirostrum dubium* (also referred to as finch-billed mynas). Like the pale-bellied myna, this is a species from Sulawesi, that is traded in substantial numbers outside its native range and that has established itself on Java and Indonesian Borneo [57,58,59]. Research on the extent of which non-native invasive mynas have established populations throughout Indonesia and how they interact with the local resident bird community is still in its infancy. 

## 5. Conclusions and Future Prospects

At present, effective management of, and regulation of trade in, invasive non-native birds in Indonesia falls short and inadvertently greatly aids both their introduction and establishment. A cooperative relationship between government agencies at various levels, civil society and local NGOs needs to be set up to implement an effective management plan for dealing with invasive non-native species such as mynas. It is clear that this needs to be implemented in several parts of Indonesia following a three-step process: (1) curbing the sale of invasive non-native mynas in the animal markets, if indeed any regulations are violated (sale of wild-caught birds, sale of protected species without proper permits, etc.), (2) the monitoring and early-warning of uninvaded areas, especially where they are adjacent to invaded areas such as parts of western and eastern Java, southern Bali and northern Lombok; and (3) ceasing of the release of confiscated mynas by the Indonesian authorities into areas where they do not occur naturally. 

This plan and following practices are likely implemented most effectively through a series of integrated measures at the municipality level that is then aided by other agencies and organisations. Preparation of technical guidelines for investigation, monitoring and assessment, and prevention and control may be needed to support this. These guidelines need to be shared with the widest possible number of stakeholders including the authorities tasked with seizing wildlife, commercial breeders of mynas, importers of mynas and traders and consumers in the animal markets.

## Figures and Tables

**Table 1:** Invasive and potentially invasive mynas (*Acridotheres* spp.) surveyed in trade in animal markets in Java, Bali and Lombok, outside their native range. Survey effort refers to the number of monthly surveys that were conducted in areas outside the native range of each species, and this may differ between species depending on whether or not they occur in parts of the study area.

Species	Native Range	Survey Region for Invasive Species	Survey Effort (Number of Markets)
Bank myna (*A. ginginianus*)	South Asia	Java, Bali, Lombok	241 (28)
Black-winged myna (*A. melanopterus*)	Java minus easternmost part	Easternmost Java, Bali, Lombok	21 (5)
Common myna (*A. tristis*)	Asia, incl. mainland SE Asia	Java, Bali, Lombok	241 (28)
Crested myna (A. *cristatellus*)	China, Indochina	Java, Bali, Lombok	241 (28)
Grey-rumped myna (*A. tricolor*)	Bali, Lombok	Java	226 (25)
Javan myna (*A. javanicus*)	Java and Bali	Lombok	3 (1)
Pale-bellied myna *(A. cinereus*)	South Sulawesi	Java, Bali, Lombok	241 (28))

**Table 2:** Markets surveyed for mynas (*Acridotheres* spp.), between August 2016 and February 2020, showing mean number and total number in brackets, ranked by the total mean number of invasive non-native species recorded. Included are only the numbers observed in markets that are situated in areas outside the species’ native range (a hyphen indicates that the species is native to that region). N = number of monthly surveys javan = *A. javanicus*; melan = *A. melanoptherus*; trico = *A. tricolor*; ciner = *A. cinereus*; gingi =*A. ginginanus*; trist = *A. tristis*; cris = *A. cristatellus*.

Market, City (Number of Surveys)	*javan*	*melan*	*trico*	*ciner*	*gingi*	*trist*	*crist*	Mean
Pramuka, Jakarta (14)	-	-	0	0	0.4 (6)	174.3 (2441)	9.5 (133)	184.3
Depok, Surakarta, C. Java (6)	-	-	0	0	0	142.0 (852)	0	142.0
Mataram, Lombok (4)	100.5 (402)	10.5 (42)	-	0	0	3.0 (12)	0	115.0
Jatinegara, Jakarta (10)	-	-	0	0	0	33.7 (337)	1.1 (11)	34.8
Pasty, Yogyakarta (6)	-	-	0	0	0	23.3 (140)	0.8 (5)	24.2
Sukahaji, Bandung, W. Java (23)	-	-	1.3 (29)	0	0	19.2 (443)	2.9 (66)	23.4
Plered, Cirebon, W. Java (17)	-	-	0.6 (10)	0	0	21.0 (357)	0.4 (7)	22.0
Karimata, Semarang, C. Java (7)	-	-	0	0	0	15.0 (105)	2.0 (14)	17.0
Pon, Semarang, C. Java (3)	-	-	0	0	0	11.0 (33)	0.7 (2)	11.7
Kerkhof, Garut, W. Java (35)	-	-	0	0	0	10.7 (374)	0.8 (29)	11.5
Splindit, Malang, E. Java (3)	-	-	0	0	0	9.7 (29)	0.3 (1)	10.0
Bratang, Surabaya, E. Java (3)	-	-	0	0	0	6.0 (18)	2.0 (6)	8.0
Cikurubuk, Tasikmalaya W Java (21)	-	-	0	0	0	7.5 (157)	0.1 (1)	7.5
Kupang, Surabaya, E. Java (3)	-	-	0	1.0 (3)	0.7 (2)	2.7 (8)	2.7 (8)	7.0
Mawar, Garut, W. Java (26)	-	-	0.5 (13)	0	0.1 (1)	5.7 (149)	0.3 (7)	6.5
Probolinggo, E. Java (3)	-	4.7 (14)	0	0	0.3 (1)	1.3 (4)	0	6.3
Satria, Denpasar, Bali (7)	-	4.7 (33)	-	0	0.3 (2)	1.3 (9)	0	6.3
Beringkit, Denpasar, Bali (5)	-	0.8 (4)	-	0	0	4.2 (21)	0	5.0
Plenongan, Depok, W. Java. (3)	-	-	0	0	0	4.0 (12)	0	4.0
Barito, Jakarta (15)	-	-	0	0	0	2.8 (42)	0.6 (9)	3.4
Pasundan, Sukabumi, W. Java (4)	-	-	0	0	0	1.5 (6)	0.5 (2)	2.0
Bayongbong, Garut, W. Java (3)	-	-	0	0	0	2.0 (6)	0	2.0
Rawa Lumbu, Bekasi, W. Java (3)	-	-	0	0	0	1.0 (3)	0	1.0
Pasirhayam, Cianyur, W. Java (3)	-	-	0	0	0	1.0 (3)	0	1.0
TanjakanEmpang, Bogor, W. Java (6)	-	-	0.3 (2)	0	0	0.3 (2)	0	0.7
Turi, Surabaya, E. Java (3)	-	-	0	0	0	0.7 (5)	0	0.7
Bondowoso, E. Java (3)	-	0.7 (2)	0	0	0	0	0	0.7
Merdeka, Bogor, W. Java (8)	-	-	0	0	0	0.3 (2)	0	0.3
Cipinang, Jakarta (3)	-	-	0	0	0	0.3 (1)	0	0.3
Kebayoran Lama, Jakarta (4)	-	-	0	0	0	0	0	0
**Total outside range**	402	95	74	3	12	5569	301	

## Data Availability

All data are presented in the paper. Additional data are available on request from the corresponding authors.
